# Investigation of the Influence of Temperature and Humidity on the Bandwidth of an Accelerometer

**DOI:** 10.3390/mi12080860

**Published:** 2021-07-22

**Authors:** Haoyu Huang, Weidong Fang, Chen Wang, Jian Bai, Kaiwei Wang, Qianbo Lu

**Affiliations:** 1 State Key Laboratory of Modern Optical Instrumentation, Zhejiang University, Hangzhou 310027, China; huanghaoyu@zju.edu.cn (H.H.); fangwd@zju.edu.cn (W.F.); wangkaiwei@zju.edu.cn (K.W.); 2 Department of Electrical Engineering, University of Leuven, 3001 Leuven, Belgium; chen.wang@esat.kuleuven.be; 3 Frontiers Science Center for Flexible Electronics (FSCFE), Shaanxi Institute of Flexible Electronics (SIFE) & Shaanxi Institute of Biomedical Materials and Engineering (SIBME), Northwestern Polytechnical University, Xi’an 710072, China

**Keywords:** accelerometer, bandwidth, temperature, humidity, finite element method, compensation

## Abstract

Bandwidth is an important parameter for accelerometers, in some cases, even surpassing sensitivity. However, there are few studies focused on the relationship between bandwidth and environmental conditions in practical application of accelerometers. In this paper, we systematically analyze the influence of environment on the bandwidth of accelerometers, obtaining the amplitude–frequency response curves versus damping ratio and properties of materials, wherein temperature and humidity were found as the two dominant factors that influence the bandwidth of accelerometers. Common temperature and humidity variations can result in bandwidth degradation of about 25% according to our theoretical analysis. The finite element method (FEM) is introduced to verify our theoretical analysis, and the accordance of the FEM simulation results and the theoretical results confirmed the validity of our analysis. Furthermore, a modification design is proposed to compensate for the influence of temperature and humidity on the bandwidth of accelerometers. By choosing materials with an appropriate Young’s modulus and coefficient of thermal expansion, the degradation of the bandwidth was substantially diminished by more than one order of magnitude, which can serve as a strong guide for the future realization of accelerometers with a steady and large bandwidth.

## 1. Introduction

Accelerometers are widely applied in many application fields [1], such as inertial navigation systems [2], seismic monitoring [3], gesture detection [4], vibration measurement [5], and gravity gradient measurement [6]. Sensitivity and bandwidth are two incompatible requirements of an accelerometer, and the former one always attracts more attention. However, in some specific areas, the bandwidth is more important [7], and lower sensitivity can be acceptable [8]. In general, effective bandwidth means the deviation between the actual response and theoretical response of an analog accelerometer is less than 3 dB [9].

**Figure 1:**
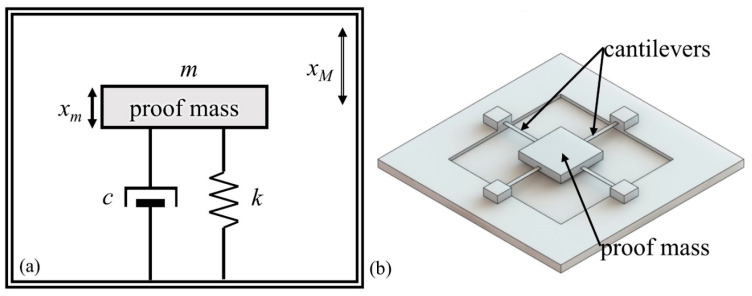
(**a**) theoretical model and (**b**) schematic diagram of the vibration structure of an accelerometer.

Thus, as the characterization of bandwidth, the amplitude–frequency response curve of accelerometers is necessary to investigate. The vibration model of an accelerometer can be simplified into a damped simple harmonic oscillator system model containing the spring and damper, as shown in Figure 1a. The proof mass, spring, and damper all influence the vibration of the accelerometers. Referring to previous research, a parameter called the damping ratio greatly determines the dynamic performance of an accelerometer, and its best value is 0.7, wherein the bandwidth of an accelerometer equals the first-order resonant frequency [10]. Clearly, variation of the damping ratio will result in a decrease of the bandwidth, while systematic research has remained elusive.

The damping ratio is correlated to the proof mass, spring, and damper. A schematic diagram of the vibration structure of an accelerometer is depicted in Figure 1b. The proof mass *m* is the vibrated structure suspended by the cantilevers, and the elastic coefficient *k* of cantilevers depends on the parameters. In most MEMS devices, the air damping is the primary source of the damping coefficient *c* in the model [11]. In practice conditions, the damping ratio involves the manufacturing errors, the deviation between theories and reality, as well as environmental factors.

As the former two are difficult to adjust in actual use, we focus on the impact of the environment, which has not been clearly formulated. Different environment conditions significantly affect the performances of accelerometers, including the bandwidth and linearity of the displacement response. For example, the temperature-varied Young’s modulus and the coefficient of thermal expansion of the materials led to temperature-varied elastic coefficients of springs [12].

In addition, the squeeze-film air damping coefficient depends on the dimensions of the structures and the proprieties of materials, which are influenced by the environment, especially the temperature and humidity. Therefore, the environment changes the displacement response and, thus, the bandwidth through the squeeze-film air damping. Squeeze-film air damping has been widely investigated. The air film was treated as a combination of a spring and a damper by Blech [13]. The effects of pressure and vibration frequency on the air damping parameters for an oscillating resonator were observed separately by Yang et al. [14]. However, these studies did not investigate the impact of temperature and humidity.

The aim of this paper is to investigate the impact of temperature and humidity on the bandwidth of accelerometers. In Section 2, a theoretical model that can characterize the vibration of accelerometers and the effect of temperature and humidity on bandwidth is introduced, wherein the dynamic response is quantitively analyzed to extract that the temperature and humidity are two dominant factors. In Section 3, a simulation model, including solid geometry and fluid geometry, is built to verify the reliability of the theoretical analysis. In addition, a method to diminish the effect of temperature on bandwidth is proposed based upon the analysis. Finally, discussions and conclusions are provided in Section 4, and we propose that the aforementioned analysis and method can enable the future realization of accelerometers with a good bandwidth.

## 2. Theoretical Model

### 2.1. Vibration Model and Bandwidth of Accelerometer

Acceleration is always measured by the displacement of the proof mass, whereas the bandwidth depends on the displacement amplitude–frequency response of the system. As shown in Figure 1, the accelerometer can be regarded as a spring-damping system, and the displacement of the entire device is:

(1) $$x_{M}=d_{0}\sin\omega t$$

where *d*_0_ and *ω* are the amplitude and frequency of vibration, respectively. The displacement of the proof mass is supposed as *x_m_*; then, the second-order differential equation of the displacement of the proof mass can be written as:

(2) $$m\ddot{x}+c({\dot{x}}_{m}-{\dot{x}}_{M})+k(x_{m}-x_{M})=0,$$

where *m* is the weight of the proof mass, *c* is the damping coefficient, and *k* is the elastic coefficient of cantilevers. We term the relative displacement between the proof mass and entire device as *z = x_m_ − x_M_*, which is the displacement actually measured, and then Equation (2) turns into:

(3) $$m\ddot{z}+c\dot{z}+kz=-m{\ddot{x}}_{M}=md_{0}\omega^{2}\sin\omega t=ma_{0}\sin\omega t,$$

where *a*_0_ is the equivalent acceleration experienced by the proof mass, equal to *d_0_ω*^2^. Omit the process of the solution, and we obtain the relative displacement of the proof mass under external vibration as:

(4) $$z=\frac{a_{0}}{\sqrt{{\left(\omega_{0}^{2}-\omega^{2}\right)}^{2}+4n^{2}\omega^{2}}}\sin(\omega t-\tan^{-1}\frac{2n\omega }{\omega_{0}^{2}-\omega^{2}}),$$

where *ω*_0_ = *√k/m* is the resonant frequency of the accelerometer, and *n* = *c*/2*m* is a factor related to the damping coefficient. Normalize the amplitude of the displacement response, and we obtain the final expression of the normalized response amplitude as:

(5) $$\beta =\frac{z}{z_{0}}=\frac{1}{\sqrt{{\left(1-\frac{\omega^{2}}{\omega_{0}^{2}}\right)}^{2}+4\frac{n^{2}}{\omega_{0}^{2}}\frac{\omega^{2}}{\omega_{0}^{2}}}}=\frac{1}{\sqrt{{\left(1-\lambda^{2}\right)}^{2}+4\zeta^{2}\lambda^{2}}},$$

where *z*_0_ = *ma*_0_*/k* is the ideal response amplitude of the proof mass, *λ* = *ω*/*ω*_0_ is the normalized frequency, and *ζ* = *n*/*ω*_0_ is the damping ratio.

For an ideal accelerometer, the normalized amplitude *β* should be close to 1. According to Equation (5), *β* is approximately equal to 1 when the normalized frequency *λ* << 1. It can be easily obtained that the maximum of *β* is 1/(2*ζ√*1 − *ζ*^2^) at *λ* = *√*(1 − 2*ζ*^2^) by the derivation of *β* when *λ* is approaching 1. It is clear that the resonance peak disappears when *ζ* ≥ 0.7, resulting in a large flat area in the amplitude–frequency response curve. Therefore, the damping ratio *ζ* = 0.7 is an ideal choice for accelerometers in consideration of bandwidth.

In general conditions, it is a standard for accelerometers that the deviation between the actual response amplitude and ideal amplitude response is less than 3 dB. Hence, the bandwidth can be represented by the maxim normalized frequency *λ* for the case where the normalized amplitude *β* > 0.707, which depends on the damping ratio *ζ*. Namely, the bandwidth of accelerometers is influenced by the damping ratio, which can be expressed as [15]:

(6) $$\zeta =\frac{c}{2m\omega_{0}}=\frac{c}{2\sqrt{km}}.$$

The damping ratio has a considerable influence on the amplitude–frequency response curve of accelerometers as mentioned above, and it is dictated by the damping coefficient, elastic coefficient of cantilevers, and weight of the proof mass. The proof mass is supposed to have an equal length and width, and the elastic coefficient *k*, quality *m*, and impact of temperature on the damping coefficient can be respectively expressed as:

(7) $$k=\frac{4Ebz^{3}}{x^{3}},$$

(8) $$m=\rho tl^{2},$$

and

(9) $$c=\frac{0.42\mu l^{4}}{h^{3}}\times {\left(1+\frac{8h}{3\pi l}\right)}^{4},$$

where *E* is the Young’s modulus, and *b*, *z*, and *x* are the width, thickness, and length of the cantilevers, respectively. *ρ* is the density of the material, *t* is the height of the proof mass, *l* is the length of the proof mass equal to the air film length, *μ* is the air viscosity, and *h* is the air film thickness. By substituting the expressions of *k*, *m,* and *c* into Equation (6), the damping ratio can be expressed as:

(10) $$\zeta =\frac{c}{2\sqrt{km}}=\frac{0.42\mu l^{4}}{2h^{3}\sqrt{\frac{4Ebz^{3}}{x^{3}}m}}\times {\left(1+\frac{8h}{3\pi l}\right)}^{4}.$$

Thus, Equation (10) clearly shows that the damping ratio of the accelerometers is affected by the air viscosity, air film thickness, dimensions of the proof mass, and cantilevers. Next, we will discuss the influence of temperature and humidity in these parameters in detail to show the influence of them on the bandwidth of accelerometers.

### 2.2. Environment Effect on Bandwidth of Accelerometer

From above analysis, we know that the bandwidth of an accelerometer is related to the damping ratio, which is mainly affected by the weight of the proof mass, the damping coefficient, and the elastic coefficient of spring. More specifically, it is influenced by the air viscosity, Young’s modulus, and coefficient of thermal expansion of materials, and dimensions of the proof mass and cantilevers. Thus, the environmental impact on the bandwidth of accelerometers can be readily adjusted by changing these parameters.

**Figure 2:**
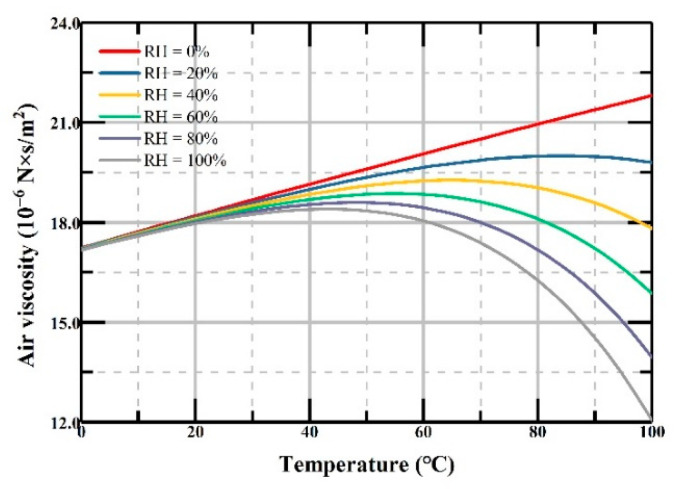
The air viscosity versus temperature with different relative humidity values.

First, the thermophysical properties of humid air have been analyzed mathematically with different temperature and humidity in previous research [16]. The varied air viscosity was calculated by a complex equation set, with parameters including dry air and water vapor viscosity, temperature with units of Kelvin and Celsius, and many measured numbers and fitting functions. The curves of air viscosity with different relative humidity *RH* versus temperature are shown in Figure 2. In order to further simplify the calculation, we fit the varied air viscosity curves shown in Figure 2 with polynomial equations:

(11) $$\begin{array}{l} \mu_{T}=\left(17.23360-0.06714\times RH\right)+\left(0.04945-0.00328\times RH\right)T\\ \;+\left(-4.23667-27.69924\times RH+1.47063\times RH^{2}\right)\times 10^{-5}T^{2}\\ \;+\left(0.601+12.80069\times RH-5.28249\times RH^{2}\right)\times 10^{-7}T^{3}\\ \;+\left(-0.00615-8.24316\times RH+0.76169\times RH^{2}\right)\times 10^{-8}T^{4} \end{array}$$

where *RH* and *T* are the relative humidity and temperature of the air.

It is clear that the air viscosity changes when the temperature *T* and relative humidity change. When the relative humidity is 0%, the air viscosity continues rising from 0 to 100 °C, and vice versa. The air viscosity first increases and then remains flat when the relative humidity is 20%. When the relative humidity becomes 40% or higher, the air viscosity will first increase and then decrease as the temperature goes up. Combining Equations (10) and (11), we determined that the damping ratio changes from −29.8% to +26.6% when the temperature varies between 0 and 100 °C with different relative humidity values.

The deviation of the normalized amplitude response between the ideal normalized amplitude response should be smaller than 3 dB in the bandwidth in actual design. Herein, we take the ideal damping ratio of 0.7 at 0 °C as the initial state. According to Equation (10), the damping ratio is related to the air viscosity, which changes with the temperature and relative humidity. Considering the temperature and relative humidity that we take, the changes of the air viscosity are shown in Figure 2.

**Figure 3:**
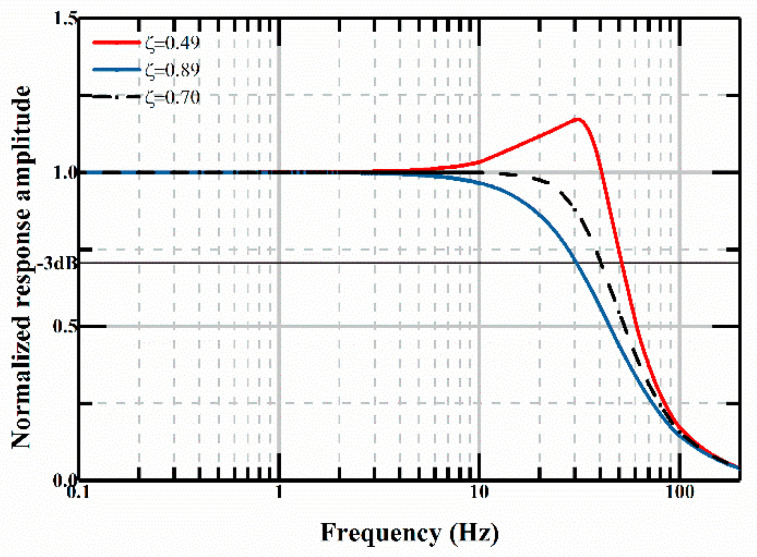
Normalized response amplitude versus frequency when *ζ* = 0.49, 0.7 and 0.89.

By substituting the air viscosity in Equation (10), the damping ratio changes from 0.49 to 0.89 under different temperature and relative humidity. By substituting the standard deviation into Equation (5), the calculated bandwidth of accelerometers goes down to 76% of the initial value, as shown in Figure 3. The red line, black dotted line, and blue line are the normalized response amplitude versus frequency when the damping ratio is 0.49, 0.70, and 0.89, respectively. Its linearity of response is greatly reduced at the same time.

When the damping ratio is perfectly 0.7, as depicted as the dashed dot line in Figure 3, the normalized response amplitude maintains constant until the frequency is larger than 10 Hz, then it will slowly decrease. However, when the damping ratio goes down, as shown in the red curve, the normalized response amplitude will first increase smoothly in the frequency region from 5 to 30 Hz, and then decrease rapidly when the frequency is around 30 Hz. When the damping ratio is higher, as shown in the blue curve, the normalized response amplitude will decrease when the frequency is around 5 Hz and decrease faster than the case where the damping ratio is 0.7.

**Figure 4:**
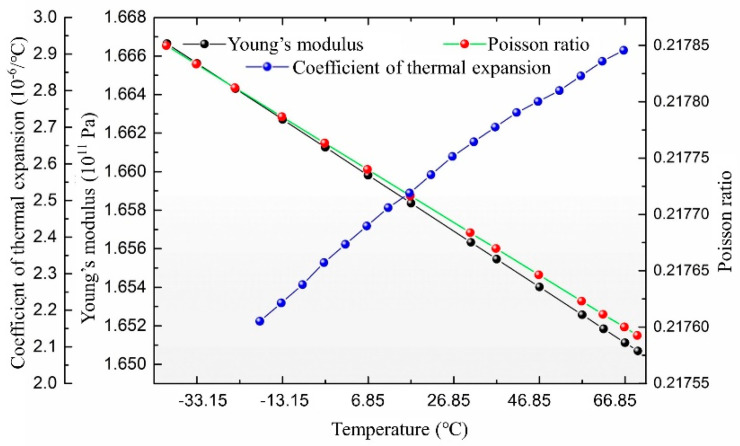
The curve of Young’s modulus, Poisson’s ratio, and the coefficient of thermal expansion of silicon versus temperature [17].

Compared with the air viscosity, temperature has very little impact on the damping coefficient in terms of the dimensional change of structures. According to Equation (9), the damping coefficient is related to the thickness and length of the air film, and temperature variation will slightly change the thickness and length of air film through thermal expansion and contraction of geometry. However, this change is small enough to be ignored considering the small thermal expansion coefficient of conventional materials, such as silicon, as shown in the blue dot line in Figure 4.

Finally, referring to Equation (7), the elastic coefficient of the spring *k* is calculated by Young’s modulus *E* and the cantilever’s dimensions, such as the length *x*, width *b*, and thickness *z*, which are all affected by temperature. The change of Young’s modulus with temperature is shown as the black dots in Figure 4, which is less than 0.5% with a temperature change of 30 °C and can be ignored compared with the influence of air viscosity. In addition, the dimensions of the cantilever also vary little. In summary, according to the material’s properties, the elastic coefficient of cantilever varies by 0.6% when the temperature increases from 20 to 50 °C, which is small enough to ignore.

## 3. Simulation Verification

FEM is a numerical method that has been proven to deal with the simulation of performance of the sensitive structure and fluid with high accuracy. Thus, it is used to verify the correctness of our theoretical analysis in this section. In addition, a compensation method is provided to diminish the effect of temperature and humidity based on the analysis.

### 3.1. Simulation Model

**Figure 5:**
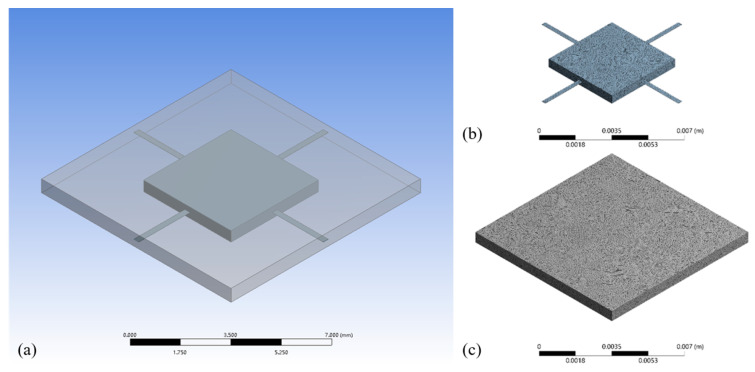
(**a**) geometric model of the sensitive structure and fluid geometry; (**b**) meshed sensitive structure; and (**c**) meshed fluid geometry.

The schematic along with the meshed geometries of simulation are shown in Figure 5. The non-transparent geometry is the sensitive structure composed of the proof mass and cantilevers, whose meshed result is shown Figure 5b. The transparent geometry surrounding the sensitive structure is the fluid, and its meshed result is shown in Figure 5c. More detailed parameters of these two geometries are listed in Table 1. The designated damping ratio is 0.7, which is the ideal case for accelerometers.

The squeeze-film air damping is the energy loss of the structure’s vibration caused by the intersection of the vibrating plate and gas. The intersection is generally expressed as a damping force *F_d_ = cv*, where *c* is the damping coefficient, and *v* is the velocity of the plate. Considering Equation (2), the first order differential term (damping term) can be completely replaced by the squeeze-film air damping force. Thus, the damping coefficient of the vibration structure under different environments can be determined by the expression of the damping force, which can be obtained by simulating the intersection of the vibrating plate and gas under different environments.

**Figure 6:**
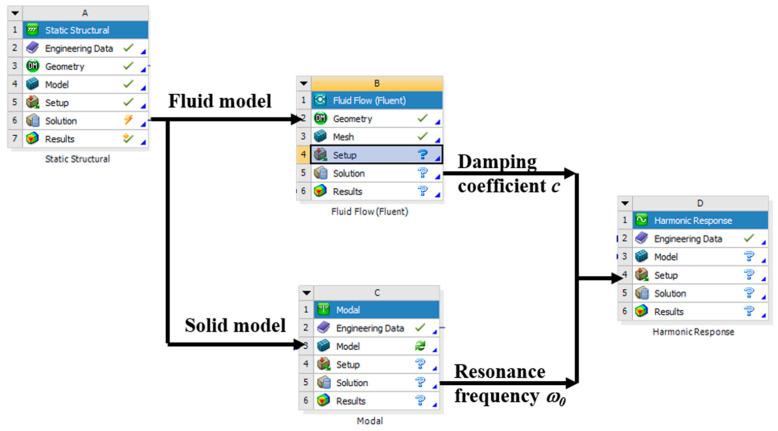
Flow diagram of the simulation.

As shown in Figure 6, the geometry was first simulated in the Static Structure module to obtain the results of structural deformation with different ambient temperatures. Then, the geometry of fluid was imported into the Fluent module. Air was chosen as the material of fluid, and the air viscosity was set as Equation (11) with different temperature and relative humidity. The squeeze-film air damping coefficient *c* of the plate vibration with a small amplitude was simulated in this module.

Next, the deformed geometry of sensitive structure was imported into the Modal module, and silicon was chosen as the material. The resonant frequency of the vibrating structure was simulated with different temperatures and properties of the material. Finally, the geometry of the sensitive structure and damping ratio obtained with the damping coefficient and resonant frequency were both imported into the Harmonic Response module to obtain the amplitude–frequency response curve.

### 3.2. Benchmark between the Simulation and Theoretical Results

#### 3.2.1. Damping Coefficient

**Figure 7:**
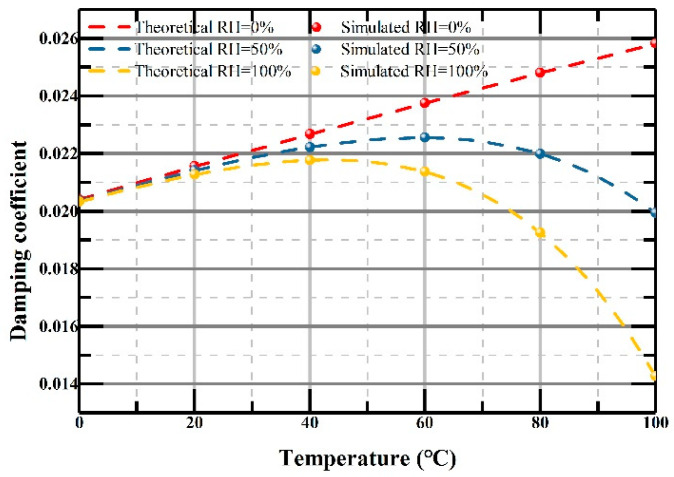
damping coefficient of the vibrating structure versus temperature under the conditions of relative humidity at 0%, 50%, and 100%.

In order to improve the efficiency of the simulation, we choose three relative humidity values: 0%, 50% and 100%. The curves of the damping coefficient versus temperature with different relative humidity values are shown in Figure 7, in which the dashed lines represent the theoretical damping coefficients, while the dots represent damping coefficients obtained by simulation in different environmental conditions. The good coincidence between them fully confirms the validity of the theoretical analysis of squeeze-film air damping and air viscosity in Section 2.2.

#### 3.2.2. Vibration Response

**Figure 8:**
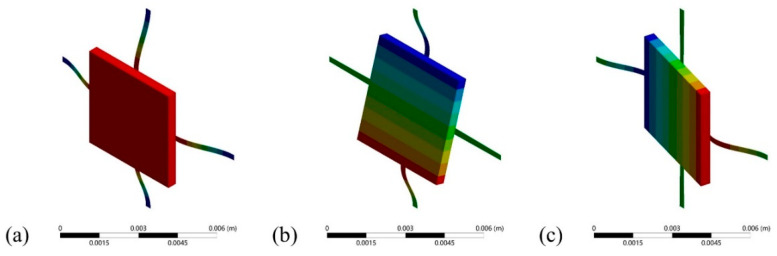
The simulation results of modal analysis for the structure for (**a**) first-order resonant mode; (**b**) second-order resonant mode; and (**c**) third-order resonant mode.

The results of the modal analysis for the structure are shown in Figure 8, where (a), (b), and (c) are the simulation results for the first-order, second-order, and third-order modules, respectively.

**Figure 9:**
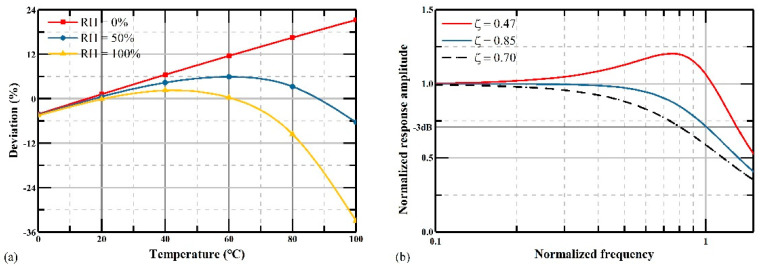
(**a**) error of the damping ratio between the simulation and design; and (**b**) normalized amplitude–frequency response curve when *ζ=* 0.47, 0.7, and 0.85.

The first-order resonant frequency of the sensitive structure obtained through the simulation is approximately equal to 115.32 Hz, which is slightly higher than the designated value. The reason of the deviation may be caused by an asymmetric mesh of the sensitivity structure. The simulation results of the damping ratio that we obtained under different environmental conditions are shown in Figure 9a.

These results show the curve of deviation between the damping ratios of different environments and the designated damping ratio 0.70, whose trend is highly consistent with that of air viscosity. According to Equation (6), by substituting the damping coefficient *c* and resonant frequency *ω_0_* obtained from simulation, the damping ratio changed from 0.47 to 0.85 with different temperatures and relative humidity values, and is in excellent agreement with the theoretical results in Section 2.2. The basic damping ratio of the simulated geometry at 0 °C was slightly lower than the designed value, which was mainly due to the higher resonant frequency in the simulation.

Referring to the previous analysis, changes of the damping ratio will change the amplitude–frequency response curve of the accelerometers. By substituting the damping ratio into the Harmonic Response module, the amplitude–frequency response curves of different conditions were obtained, as shown in Figure 9b. These results indicate that the actual amplitude–frequency response curves of the structure with the maximum and minimum damping ratio had larger deviations compared to the perfect one.

The bandwidth of the accelerometer was reduced from 100% to 78% according to the 3 dB standard, and the linearity of the response amplitude also decreased severely. Therefore, the simulation results explicitly show the correctness of our theoretical analysis of the impact on the bandwidth of accelerometers, and the impact could be considerable enough to require elimination.

#### 3.2.3. Compensation Guide for Bandwidth under Environment Effect

As mentioned above, temperature and humidity are two dominant environment factors involving the damping ratio of a sensitive structure, and this ratio then influences the amplitude–frequency response of the accelerometer and, finally, the bandwidth. The humidity is commonly hard to adjust with a specific package. Although the temperature can be stabilized by temperature control, other components, such as the heating resistance, temperature measuring resistance, and control circuit, are inevitably introduced. Hence, it is better to compensate the temperature effect via structural design. A compensation guide is shortly included in this subsection.

According to the theoretical model, the Young’ modulus and coefficient of thermal expansion of the materials have an impact on the amplitude–frequency response of accelerometers; however, the impact in terms of the damping ratio is typically ignored because conventional materials are of little change with temperature in these two characteristics. However, there is no doubt that the damping ratio will be highly influenced if these two characteristics of materials change with temperature.

From the above analysis, the damping ratio should be a constant if we want to maintain the bandwidth of the accelerometers. According to Equation (10), the damping ratio under different temperatures is dependent on the air viscosity *μ*, the Young’s modulus, and dimensions of the proof mass and cantilevers. Thus, the influence of temperature on the air viscosity can be compensated by choosing materials with an appropriate Young’s modulus or coefficient of thermal expansion, and the bandwidth of the accelerometers can, thus, be maintained. To ensure the bandwidth of accelerometers remains unchanged with varied temperatures, the damping ratio of the structure should be a constant under different temperatures:

(12) $$\begin{array}{l} \zeta_{T}=\frac{0.42\mu_{T}l_{T}{}^{4}}{2h_{T}{}^{3}\sqrt{\frac{4E_{T}b_{T}z_{T}{}^{3}}{x_{T}{}^{3}}m}}\times {\left(1+\frac{8h_{T}}{3\pi l_{T}}\right)}^{4}\\ =\frac{0.42l_{0}{}^{4}}{2h_{0}{}^{3}\sqrt{\frac{4b_{0}z_{T}{}^{3}}{x_{0}{}^{3}}m}}\times {\left(1+\frac{8h_{0}}{3\pi l_{0}}\right)}^{4}\times \frac{\mu_{T}\sqrt{1+CTE_{T}\times T}}{\sqrt{E_{T}}}=constant \end{array}$$

where *E_T_* is the Young’s modulus under temperature *T*, and *b_0_*, *z_0,_* and *x_0_* are the initial width, thickness, and length of the cantilevers under temperature 0 °C respectively. *b_T_*, *z_T,_* and *x_T_* are the width, thickness, and length of the cantilevers under temperature *T*, respectively. *l_0_* and *l_T_* are the length of the proof mass under the temperatures 0 °C and *T,* respectively, equal the air film length. *μ_T_* is the air viscosity under temperature *T*. *h_0_* and *h_T_* are the air film thickness under the temperatures 0 °C and *T,* respectively. *CTE_T_* is the coefficient of thermal expansion under temperature *T*.

Based on the above discussion, for the materials w the coefficient of thermal expansion is small enough to be ignored, the Young’s modulus *E_T_* of materials should be proportional to the square of the air viscosity *μ_T_* under temperature *T* to maintain the damping ratio of accelerometers:

(13) $$E\propto \mu_{T}{}^{2}.$$

For the materials that the Young’s modulus is small enough to be ignored, the coefficient of thermal expansion *CTE_T_* and the air viscosity *μ_T_* should satisfy:

(14) $$CTE_{T}=\frac{\left[{\left(\frac{\mu_{0}}{\mu_{T}}\right)}^{2}-1\right]}{T}.$$

The effect of temperature can be compensated if we use materials with an appropriate Young’s modulus or coefficient of thermal expansion.

**Figure 10:**
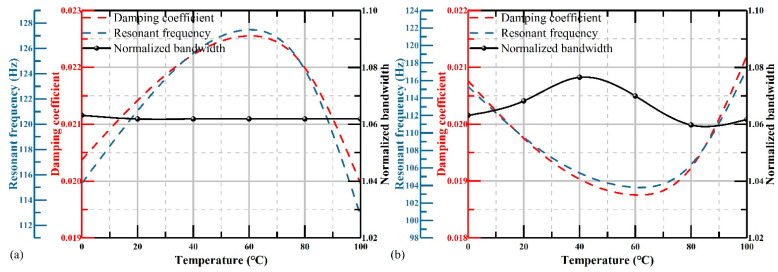
The resonant frequency, damping coefficient, and damping ratio versus the temperature of compensation with the (**a**) Young’s modulus and (**b**) coefficient of thermal expansion.

To verify the correctness of our analysis, adopting simulations based on the Young’s modulus and the coefficient of thermal expansion, which were determined above, is appropriate. The relative humidity is supposed as a constant in the simulation, and here we take it as 50%. The temperature varies from 0 °C to 100 °C. Substituting *RH* = 50% into Equation (11), we obtain the air viscosity. Then, by substituting the air viscosity into Equations (13) and (14), the simulation results that satisfy the Young’s modulus only and the coefficient of thermal expansion only are shown in Figure 10a and b, respectively.

The changes of resonant frequency of the sensitive structure, damping coefficient, and bandwidth of the accelerometer are shown as the blue dotted line, red dotted line, and black dots, respectively. It is clear that the resonant frequency in both Figure 10a,b changed with temperature and had the same trend as the damping coefficient. As a result, the bandwidth was almost unchanged with the temperature. The change in bandwidth was reduced from 25% to 0.15% and 1.5%, respectively by choosing materials that satisfied the Young’s modulus and the coefficient of thermal expansion.

This shows the effectiveness of the compensation method. The damping ratio of compensation for Young’s modulus varied less compared to the coefficient of thermal expansion. This was mainly due to the coefficient of thermal expansion changing the damping coefficient and resonant frequency together, which introduced more errors than the Young’s modulus.

In practice, the Young’s modulus of rubber materials is proportional to the temperature, such as with SiC [18]. If we take rubber as the material of the cantilevers, the changes in bandwidth reduce from 25% to 3.6% under the 50% relative humidity from 0 to 100 °C. This fully illustrates that, even being unable to find materials that fully meet the compensation requirements, materials with similar properties can also compensate for the influence of temperature on the bandwidth.

## 4. Discussion and Conclusions

This paper investigated the bandwidth of accelerometers through analyzing the amplitude–frequency response curve, damping ratio, and properties of materials with different environmental conditions. We found that changes in the temperature and relative humidity reduced the bandwidth of accelerometers by about 25% by changing the air viscosity, while their influences on the Young’s modulus and coefficient of thermal expansion of conventional materials were small enough to be ignored. FEM was used to verify the correctness of our theoretical analysis.

Good agreement between them was observed, confirming the validity of the theoretical analysis. Furthermore, a compensation method by utilizing materials with a Young’s modulus and coefficient of thermal expansion appropriate for temperature change was proposed based on the theory of the effect of temperature and humidity on the bandwidth of accelerometers. The results of the method showed that the change in bandwidth reduced from 25% to less than 1.5% by choosing materials with an appropriate Young’s modulus and coefficient of thermal expansion, which holds the promise of effective and convenient compensation. Although it is difficult to find perfectly designated materials, the compensation can still be partly achieved by choosing materials with similar properties. Moreover, the compensation result can be even better when taking the Young’s modulus and coefficient of thermal expansion into consideration together.

In summary, the influence of temperature and humidity on the bandwidth of accelerometers was systematically analyzed in this work, and the temperature and humidity were concluded to be the two main factors that affect the bandwidth. In addition, a compensation guide was introduced and showed good results in compensating the bandwidth under temperature effects. This work provides a direct reference for the bandwidth changes of accelerometers under different temperatures and humidity values and paves the way for the future design of accelerometers with a steady and large bandwidth.

## Figures and Tables

**Table 1:** Numerical value of the parameters used in our simulation.

Name	Unit	Numerical Value
Density of silicon	Kg/m^3^	2330
Young’s modulus of silicon	GPa	Refer to Figure 4
Coefficient of thermal expansion of silicon	--	Refer to Figure 4
Length of the proof mass	m	4.29 × 10^−3^
Width of the proof mass	m	4.29 × 10^−3^
Height of the proof mass	m	0.49 × 10^−3^
Length of the cantilever	m	2.50 × 10^−3^
Width of the cantilever	m	240 × 10^−6^
Height of the cantilever	m	10 × 10^−6^
Weight of the proof mass	kg	2.10 × 10^−5^
Elastic coefficient	N/m	10.20
First-order resonant frequency	Hz	110.88
Air film thickness	m	50 × 10^−6^
Air viscosity	--	Refer to Equation (11)
Designed damping coefficient		0.0204
Designated damping ratio	--	0.7

## Data Availability

Not applicable.
